# Postpartum Uterine Ultrasonographic Scale: a novel method to standardize the assessment of uterine postpartum involution

**DOI:** 10.25122/jml-2020-0107

**Published:** 2021

**Authors:** Roxana Covali, Demetra Socolov, Razvan Vladimir Socolov, Mona Akad

**Affiliations:** 1. Department of Radiology, Elena Doamna Obstetrics and Gynecology University Hospital, Iasi, Romania; 2. Department of Obstetrics and Gynecology, Cuza Voda Obstetrics and Gynecology University Hospital, Iasi, Romania; 3. Department of Obstetrics and Gynecology, Elena Doamna Obstetrics and Gynecology University Hospital, Iasi, Romania; 4. University of Medicine and Pharmacy Grigore T. Popa Iasi, Romania

**Keywords:** uterine ultrasonography, obstetric delivery, postpartum hemorrhage, uterine retraction, endometrial length

## Abstract

Postpartum hemorrhage is a leading cause of maternal mortality. Various methods can be used to evaluate the postpartum uterine cavity volume. This work aims to introduce a simple method for uterine postpartum cavity volume evaluation, called Postpartum Uterine Ultrasonographic Scale (PUUS), which could be used routinely. In this prospective study, 131 consecutive Caucasian patients were evaluated by using the PUUS method. The mean age was 27.72 years (ranging from 15 to 42). Patients were examined in the same time intervals: within the first 24-48 hours after delivery in case of vaginal delivery, and within the first 48-72 hours, in case of cesarean delivery. Patients with PUUS grades 2, 3, or 4 were reexamined daily until the PUUS grade declined to 1 or 0. The PUUS method evaluated the length of the endometrium of the uterine cavity occupied by blood or debris, from grade 0 (no blood) to grade 4 (over three-quarters of the endometrial length occupied by blood/debris). The PUUS grade of uterine involution varied with the day of examination, gestation, and parity. In this article, a novel method of evaluating uterine postpartum involution titled PUUS is introduced. This method standardized uterine cavity involution in a numerical fashion. We hope that the PUUS scale could further be used to decrease the morbidity and mortality of women due to postpartum hemorrhage.

## Introduction

Postpartum hemorrhage is a leading cause of maternal mortality worldwide [1–3]. Therefore, postpartum uterine evaluation after delivery is extremely important. Poor uterine tone accounts for about 80% of all cases of primary postpartum hemorrhage [2]. Although postpartum hemorrhage itself may not be preventable, early identification of blood loss and mobilization of resources may prevent adverse outcomes [3]. Ultrasonographic evaluation of the postpartum uterus may reveal a closed cavity, hematometra, or debris. Belachew *et al.* used 3-dimensional transabdominal ultrasound imaging to describe uterine involution in the puerperium and discovered intrauterine content in 36% of the women on day 1, 95% on day 7, and 87% on day 14 [4]. Diniz *et al.* reported that the uterine cavity was filled with some sort of material in 72.9% of the patients in the initial puerperium (initial 48 hours) whose uteri were examined through ultrasound and uterine artery Doppler [5]. There is a massive difference between 36% of women on day 1 and 72.9% of women within the first 48 hours postpartum; thus, further research is required.

Various methods can be used to evaluate the uterine postpartum cavity volume: abdominal and/or transvaginal ultrasonography, focused assessment with sonography for obstetrics, 2- and 3-dimensional ultrasound examinations, contrast-enhanced ultrasound, computed tomography (CT) scans [6], MRI [7] or even transabdominal and transvaginal sonography with magnetic resonance imaging fusion. Measurements can be made using these various approaches of the uterine cavity diameters, uterine volume, the lower uterine segment thickness, the posterior or anterior uterine wall, the cesarean scar, gas in the uterine cavity, and so on. But these approaches cannot be used routinely. In addition, there is no consensus on a standardized method for postpartum ultrasound; therefore, more research and standardization are necessary.

Hence the objectives of our study are to introduce a simple, easy method to evaluate postpartum uterine involution, to introduce a standardized method to evaluate postpartum uterine involution, to evaluate the uterine cavity volume daily during the first postpartum days. This study describes a simple method called the Postpartum Uterine Ultrasonographic Scale (PUUS), which can be used routinely.

## Material and Methods

The routine protocol in our hospital includes standard transabdominal ultrasound examination of the uterus in the first 24–48 hours after delivery in case of vaginal delivery, and in the first 48–72 hours, in case of cesarean delivery. The goal of the routine examination is to evaluate whether the uterine cavity is closed or still open. When the uterine cavity shows a small region of echogenicity, with a maximum diameter of less than 1 cm, or only the endometrium, the patient is considered within normal limits of uterine involution. When the uterine cavity holds more than 1 cm maximum diameter of blood or debris, the patient is reevaluated by abdominal ultrasound the next day. In case the content of the uterine cavity does not decrease, additional oxytocin injections, twice a day, on three successive days, are administered to the patient. Daily abdominal ultrasound is performed until the uterine content decreases to under 1 cm maximum diameter. When no improvement after oxytocin administration is noticed, uterine curettage is the only option left.

Still, this is a subjective way of evaluating the decrease in the uterine cavity, especially when following the involution of the uterine cavity of any particular patient. Therefore, a standardized way to describe and follow the involution is required.

**Figure 1.**
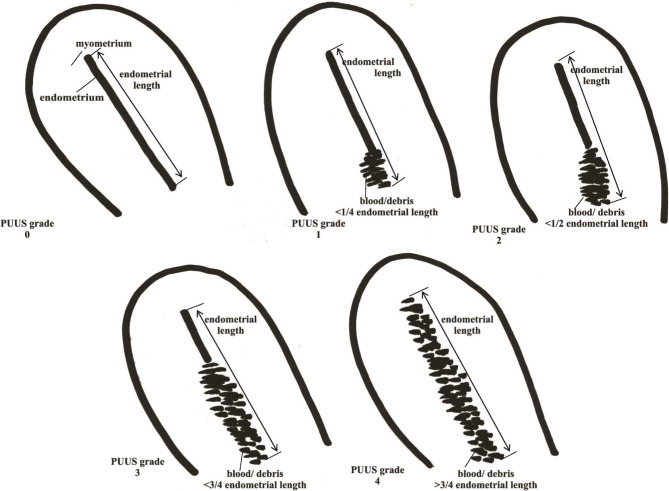
PUUS grades 0–4 on uterine ultrasonography.

A novel way of evaluation, the Postpartum Uterine Ultrasonographic Scale (PUUS), was introduced. The PUUS method evaluates the proportion of the endometrial length occupied by blood or debris, as follows:

• Grade 0: no blood or debris in the uterine cavity;
• Grade 1: less than a quarter of the endometrial length occupied by blood or debris;
• Grade 2: less than a half of the endometrial length occupied by blood or debris;
• Grade 3: less than three-quarters of the endometrial length occupied by blood or debris;
• Grade 4: over three-quarters of the endometrial length is occupied by blood or debris (Figure 1).

In this prospective study, 160 consecutive Caucasian patients, who delivered in an obstetrics and gynecology university hospital, from October 2017 until December 2017, either by cesarean delivery or by vaginal delivery, were considered for evaluation using the PUUS method. After excluding 29 patients (as described below), 131 patients were included in our study.

Inclusion criteria were as follows:

1. Patients who delivered at 34 weeks of pregnancy or more, either by cesarean delivery or by vaginal delivery;
2. Patients who delivered in our hospital;
4. Patients who had no infectious/highly contagious health problem.

Exclusion criteria were as follows:

1. Patients who delivered under 34 weeks of pregnancy;
2. Patients who delivered at home;
3. Patients who had infectious/highly contagious health problems (11 patients) and were sent to a septic department of our hospital;
4. Patients with myoma, adenomyosis or other diseases that may affect the uterine involution.

The mean age was 27.72 years (ranging from 15 to 42). The patients were examined in the same time intervals: within the first 24–48 hours after delivery in case of vaginal delivery, and within the first 48–72 hours, in case of cesarean delivery. To eliminate any potential sources of bias, patients were examined in a random order, and the practitioner was unaware of the patient’s medical history at the time of the examination. As in the routine protocol, patients with PUUS grades 2, 3, or 4 were reexamined the following day. Mobility encouragement was used and, if no uterine cavity involution was noticed, oxytocin was injected twice a day, three consecutive days, and ultrasonographic examination was performed daily until the PUUS score was 1 or 0.

Statistical analysis was performed using IBM SPSS version 18. For descriptive measures, we computed the mean, standard deviation, and minimum and maximum limits. Multiple group comparison was performed using the ANOVA technique, whereas, for two sets, the student t-test was applied. ANOVA condition of variances homogeneity was assessed using the Levene statistic, and the Tamhane post-hoc test was applied if ANOVA was not accepted. P=0.05 was considered statistically significant.

## Results

**Figure 2.**
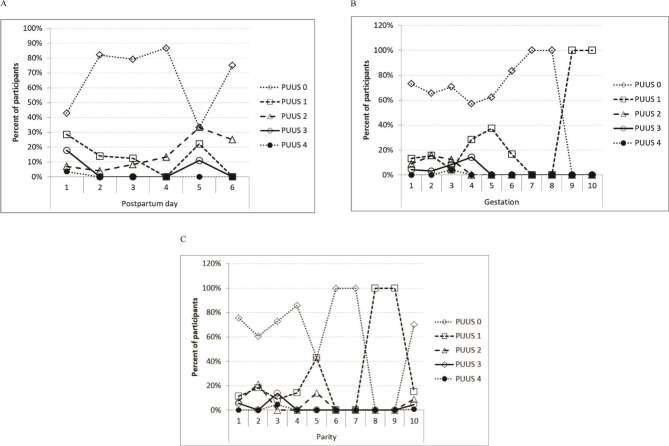
Variation of PUUS, depending on: A. Postpartum day of examination. B. Gestation and C. Parity.

During this stage of the study, no PUUS grade 4 uterus involution was found. The PUUS grade varied inversely proportionally (r=-.145, P=.099) with the day of examination (1^st^, 2^nd^, 3^rd^, 4^th^, 5^th^, 6^th^, 8^th^ day): 1.11 (±1.257), 0.22 (±0.507), 0.29 (±0.624), 0.27 (±0.704), 1.22 (±1.093), 0.5 (±1), 0 (there was only 1 case in the 8^th^ day, no standard deviation could be determined), respectively (Tables 1, 2). No patients were examined on the 7^th^ day after delivery in this study group. The authors noticed that the postpartum uterine cavity would totally collapse between 24–48h after delivery. The number of patients requiring repeated reevaluation was few, 16 out of 131 (12.21%) (Figure 2A).

**Table 1.** PUUS value depending on the postpartum day.

**Descriptives^a^**								
**Postpartum day**		**Statistic**						
		**Mean**	**95% Confidence Interval for Mean**		**Median**	**Std. Deviation**	**Minimum**	**Maximum**
			**Lower Bound**	**Upper Bound**				
**PUUS**	**1**	1.11	0.62	1.59	1.00	1.257	0	4
	**2**	0.22	0.08	0.36	0.00	0.507	0	2
	**3**	0.29	0.03	0.56	0.00	0.624	0	2
	**4**	0.27	-0.12	0.66	0.00	0.704	0	2
	**5**	1.22	0.38	2.06	1.00	1.093	0	3
	**6**	0.50	-1.09	2.09	0.00	1.000	0	2

a PUUS is constant when Postpartum day = 8. It has been omitted.

**Table 2.** Correlations between PUUS and postpartum day. There are no statistically significant correlations (P = .099), but there is a reverse correlation (r= -0.145).

**Correlations between PUUS and postpartum day**				
			**PUUS**	**Postpartum day**
**Spearman’s rho**	**PUUS**	**Correlation Coefficient**	1.000	**-.145**
		**Sig. (2-tailed)**	**.**	**.099**
		**N**	131	131
	**Postpartum day**	**Correlation Coefficient**	-.145	1.000
		**Sig. (2-tailed)**	.099	.
		**N**	131	131

The PUUS grade varied with the gestation (IG, IIG, IIIG, IVG, VG, VIG, VIIG, VIIIG, IXG, and XG): 0.44, 0.51, 0.7, 0.71, 0.55, 0.16, 0, 0, 1, 1, respectively (Tables 3, 4). There was no patient over XG in this study group. Multiple gestations exhausted the uterine muscle so that postpartum involution required a longer time, and PUUS grade was higher during the first postpartum days in patients with multiple gestations. However, there was no statistical correlation between PUUS grade and gestation (P= .957). Still, the PUUS grade slowly decreased to 1 or 0 in all patients of this group (Figure 2B).

**Table 3.** PUUS value depending on gestation.

**Descriptives^a^**								
**PUUS**		**Statistic**						
		**Mean**	**95% Confidence Interval for Mean**		**Median**	**Std. Deviation**	**Minimum**	**Maximum**
			**Lower Bound**	**Upper Bound**				
**Gestation**	**0**	2.73	2.31	3.15	2.00	2.022	1	8
	**1**	3.35	2.10	4.60	2.00	2.661	1	10
	**2**	1.92	1.41	2.42	2.00	0.793	1	3
	**3**	2.33	1.06	3.60	2.50	1.211	1	4

a Gestation is constant when PUUS = 4. It has been omitted.

**Table 4.** Correlations between PUUS values and gestation. There are no statistically significant correlations (P = .957).

**Correlations**				
			**PUUS**	**Gestation**
**Spearman's rho**	**PUUS**	**Correlation Coefficient**	1.000	.005
**Sig. (2-tailed)**	.	.957
**N**	131	131
**Gestation**	**Correlation Coefficient**	.005	1.000
	**Sig. (2-tailed)**	.957	.
	**N**	131	131

The PUUS grade varied with the parity (IP, IIP, IIIP, IVP, VP, VIP, VIIP, VIIIP, XP): 0.43, 0.6, 0.68, 0.14, 0.57, 0, 0, 0, 1, respectively (Tables 5, 6). There were no IXP or above patients in this study group. Numerous gestations exhausted the uterine muscle so that postpartum involution required a longer time, and PUUS grade was higher during the first days postpartum in multiparous than in uniparous patients. However, there was no statistically significant correlation between PUUS grade and parity (P= .642). Still, the PUUS grade slowly decreased to 1 or 0 in all patients of this group (Figure 2C).

**Table 5.** PUUS value depending on parity.

**Descriptives^a^**								
**PUUS**		**Statistic**						
		**Mean**	**95% Confidence Interval for Mean**		**Median**	**Std. Deviation**	**Minimum**	**Maximum**
			**Lower Bound**	**Upper Bound**				
**Parity**	**0**	2.30	1.97	2.64	2.00	1.615	1	7
	**1**	3.05	1.89	4.21	2.00	2.481	1	10
	**2**	1.92	1.23	2.61	2.00	1.084	1	5
	**3**	2.00	0.85	3.15	2.00	1.095	1	3

a Parity is constant when PUUS = 4. It has been omitted.

**Table 6.** Correlations between PUUS value and parity. There are no statistically significant correlations (P = .642).

**Correlations**				
			**PUUS**	**Parity**
**Spearman's rho**	**PUUS**	**Correlation Coefficient**	1.000	.041
		**Sig. (2-tailed)**	.	.642
		**N**	131	131
	**Parity**	**Correlation Coefficient**	.041	1.000
		**Sig. (2-tailed)**	.642	.
		**N**	131	131

## Discussion

This study included 131 consecutive Caucasian patients. It was not intentional to exclude other populations. In Romania, the vast majority of the population is Caucasian, with only very few percentages of other ethnicities.

Patients whose uterine cavities were not closed (PUUS grades 4, 3, or 2) or were almost closed (PUUS grade 1) were reexamined the following days. After the 3^rd^ day postpartum, most women examined were those whose uterus could not contract properly. That is why the PUUS grade increased during the 4^th^ and 5^th^ days postpartum. That was the moment when patients received oxytocin. The uterine cavities that did not close until grade 0 or grade 1 PUUS after 3 days of oxytocin required curettage and debris elimination. Because curettage in multiparous patients is hazardous, successive ultrasonographic examination of the uterus on successive days was preferred until the uterine cavity closed.

Paliulyte *et al.* used abdominal ultrasound to measure the uterine parameters after labor on the 1^st^, 3^rd^, 10^th^, 30^th^, 42^nd^, and 69^th^ postpartum days [8]. He reported that the endometrial cavity on the 10^th^ day was significantly wider in multiparous women and mainly echo-negative. He concluded that the involution trend in primiparous and multiparous women follows a similar pattern, though it lasts longer in multiparous women. The PUUS study evaluated the uterine cavity within the first 10 postpartum days and described it as mostly closed (PUUS grade between 1 and 0), starting with the 2^nd^ postpartum day in most patients (Figure 1). The follow-up continued only for the patients whose uterine cavity was PUUS grade 2 or above. It was no longer necessary to continue evaluation in most women beyond day 2 or 3 postpartum. Still, there was a patient whose PUUS grade was determined for the first time on the 8^th^ day postpartum because it was the first working day after her delivery.

Kristoschek *et al.* performed ultrasound examinations on days 1, 2, and 7 of the postpartum period [9]. The authors measured longitudinal, anteroposterior, and transverse uterine diameters and used them to calculate uterine volume. He also measured the thickness and length of the uterine cavity. He reported no correlation between uterine involution and parity. The PUUS method drew the same conclusion: though the higher the parity, the higher the PUUS grade seems to be, there was no statistically significant correlation between them.

Schweizer *et al.* performed suprapubic and endovaginal ultrasound examinations of asymptomatic patients 48 hours after their cesarean delivery. They found a hematic collection in 3 out of 31 patients, which measured a maximum of only 49 mm long [10]. In the current study, 40 patients were examined for the first time postpartum during the second day (24–48 hours) after cesarean delivery, and hematic/debris collection was found in 5 (12.5%) patients, out of which the PUUS grade was 2 in 1 (2.5%) patient and 1 in 4 (10%) patients.

Although ultrasound evaluation of the gynecologic conditions of the uterus is well documented, there are difficult situations (congenital uterine anomalies, for example [11]) that may require a combined endoscopic-ultrasound approach. Unlike that, this study addresses the paucity of standardized methods of evaluating the uterine cavity decrease of any particular patient.

With reference to the study objectives, we introduced a simple, standardized method to evaluate postpartum uterine involution. We demonstrated that uterine involution varied with the day of examination, gestation, and parity. This is the first step in describing the PUUS method. Further studies are required, with a more significant number of participants, in more hospitals, to evaluate the feasibility and appropriateness of the PUUS method. Since observatory-related variability may be encountered, interobserver agreement studies may be necessary to eliminate potential sources of bias.

This study considered only the dimensions of the uterine cavity, not the content, whether it was blood, debris, or empty. Information about the discharge of an intact placenta, the need for a curettage right after birth, or separate information about term/preterm deliveries were not studied, either. Future studies are required to address these issues, as well.

## Conclusion

This report introduces a novel method of evaluating uterine postpartum involution called PUUS. This method standardized uterine cavity involution in a numerical fashion. Though this method had no direct impact on patient outcomes, we hope that this PUUS scale could further be used to evaluate precisely and compare the effect of different medicines upon uterine involution to decrease the morbidity and mortality of patients due to postpartum hemorrhage.

## Acknowledgments

The authors would like to thank Mr. Lucian Boiculese, Associate Professor, Department of Statistics, “Grigore T. Popa” University of Medicine and Pharmacy Iasi, for contributing to this study.

### Ethical approval

The approval for this study was obtained from the Ethics Committee of the Elena Doamna Obstetrics and Gynecology University Hospital (approval ID: 9/September 15^th^, 2017).

### Consent to participate

Written informed consent was obtained from the patients.

### Conflict of interest

The authors declare that there is no conflict of interest.
